# Navigating the complexity of current interventional radiological approaches for distant metastases in thyroid cancer

**DOI:** 10.3389/fendo.2025.1445855

**Published:** 2025-07-11

**Authors:** Daqi Zhang, Jerry Spisani, Arianna Ceriello, Francesco Frattini, Francesco Brucchi, Gianlorenzo Dionigi, Lanlan Wan, Alessio Salvatore Angileri, Carla Colombo, Anna Maria Ierardi, Gianpaolo Carrafiello

**Affiliations:** ^1^ Division of Thyroid Surgery, The China-Japan Union Hospital of Jilin University, Jilin Provincial Key Laboratory of Surgical Translational Medicine, Jilin Provincial Precision Medicine Laboratory of Molecular Biology and Translational Medicine on Differentiated Thyroid Carcinoma, Changchun, Jilin, China; ^2^ Postgraduated School in General Surgery, University of Milan, Milan, Italy; ^3^ Division of General and Endocrine Surgery, Istituto Auxologico Italiano, IRCCS (Istituto di Ricovero e Cura a Carattere Scientifco), Milan, Italy; ^4^ Department of Pathophysiology and Transplantation, University of Milan, Milan, Italy; ^5^ Department of Anesthesia, The Second Hospital of Jilin University, Changchun, Jilin, China; ^6^ Radiology Department, Università Statale degli Studi Di Milano, IRCCS Policlinico, Milano, Italy; ^7^ Endocrine Oncology Unit, Department of Endocrine and Metabolic Diseases, Istituto Auxologico Italiano IRCCS, Milan, Italy

**Keywords:** papillary thyroid carcinoma, medullary thyroid carcinoma, follicular thyroid carcinoma, metastases, recurrence

## Abstract

The finding of liver metastases in thyroid cancer is relatively uncommon. Treatment is complex and depends on several factors, including location, number and size of lesions, associated symptoms, extrahepatic disease and patient’s condition. In this context, interventional radiological treatments have been successfully used to optimize disease control or for palliative purposes and have generally been well tolerated. However, careful selection of candidates for such procedures in a multidisciplinary context is crucial.

## Introduction

Thyroid cancer (TC) comprises a heterogeneous group of malignant diseases, including well-differentiated carcinomas such as papillary and follicular thyroid carcinoma — collectively referred to as differentiated thyroid carcinoma (DTC) — as well as more aggressive subtypes such as medullary thyroid carcinoma (MTC) and anaplastic thyroid carcinoma (ATC). These histotypes differ significantly in their biological behavior, prognosis and therapeutic strategies. DTC often takes an indolent course and responds well to radioiodine therapy, while MTC and especially ATC pose greater therapeutic challenges due to early metastasis and resistance to conventional treatments. In ATC, palliative measures usually offer only a temporary benefit, whereas in advanced DTC or MTC— - especially in oligometastatic disease — locoregional treatments can contribute to long-term disease control and symptom relief.

Liver metastases (LM) are seen in approximately 50% of patients with advanced MTC and usually present as diffuse liver involvement. In contrast, LM are rare in DTC and occur in only 0.5–3.5% of metastatic cases. Clinically, LM in DTC are often asymptomatic or associated with non-specific abdominal complaints; in rare cases, hyperfunctional metastases can lead to clinical hyperthyroidism. In MTC, LM can cause gastrointestinal symptoms, including diarrhoea, especially in advanced disease.

The treatment of LM in thyroid cancer is complex and is influenced by the tumor burden, location of the lesion, symptoms, performance status and extent of extrahepatic disease. Surgical resection may be appropriate for isolated, resectable LM, particularly in patients with limited extrahepatic spread, and is associated with improved survival. However, in diffuse bilobar involvement of the liver, which is common in MTC, surgical resection is usually not possible.

In DTC, LM often occurs in conjunction with radioiodine-refractory disease, making RAI therapy ineffective. In such cases, systemic therapy with tyrosine kinase inhibitors (TKIs) is often used. However, LM may respond less well and for a shorter time compared to other metastatic sites, such as lung lesions.

Interventional radiological treatments (IRT) have been shown to be valuable tools in the treatment of both primary and metastatic liver tumors. Techniques such as radiofrequency ablation (RFA), microwave ablation (MWA), transarterial chemoembolization (TACE) and selective internal radiation therapy (SIRT) offer local tumor control with relatively low systemic toxicity. Due to their minimally invasive nature, they are suitable for patients with limited functional reserve or contraindications to surgery. In thyroid cancer, IRT can not only serve palliative purposes, but can also help to postpone systemic therapy or treat oligoprogressive disease during TKI treatment.

It is controversial whether IRT could impair the efficacy of TKIs by altering tumor vascularization. Conversely, tumor removal by local therapy may improve the results of systemic treatment. Depending on patient-specific factors, different IRT modalities can be combined with systemic or surgical strategies to optimize treatment.

Given the rarity and complexity of LM in thyroid cancer, individualized treatment approaches within multidisciplinary teams are essential. The aim of this review is to summarize the current evidence and clinical considerations for the use of IRT in the treatment of liver metastases in thyroid cancer.

## Methods

For this review, we conducted a non-systematic literature search in the PubMed/MEDLINE, Scopus and Google Scholar databases up to March 2025. The search terms included combinations of “thyroid cancer”, “liver metastases”, “interventional radiology”, “radiofrequency ablation”, “chemoembolization”, “selective internal radiotherapy” and “tyrosine kinase inhibitors”. English-language articles were considered, including original studies, retrospective series, systematic reviews and relevant case reports. Studies were included based on their relevance to the role of interventional radiology in the treatment of liver metastases from DTC and MTC. We excluded articles that focused exclusively on anaplastic thyroid carcinoma or non-liver metastases or that lacked treatment-related outcomes. While our approach did not follow PRISMA guidelines, it aimed to ensure broad yet focused coverage of current evidence.

### Metastatic patterns vary by subtype

According to registry analyses, metastatic patterns differ significantly among thyroid cancer subtypes (1–3). Overall, 35.1% of patients developed metastases, with statistically significant variation among histotypes (P < 0.0001):

- 38.7% for follicular thyroid carcinoma
- 17.3% for papillary thyroid carcinoma
- 75.4% for anaplastic thyroid carcinoma
- 47.8% for medullary thyroid carcinoma

For papillary (79.7%), follicular (72.9%), and anaplastic (92.1%) subtypes, the most frequent metastatic site was the lung (1–3). In contrast, for the medullary subtype, liver metastases were most prevalent (81.3%), while lung involvement was observed in only 56.3% of cases (P < .0001). Bone metastases occurred in 40.6% of medullary and 15.7% of anaplastic cases (P = 0.008).

Cardiac metastases were noted in 23.7% of papillary and 19.1% of anaplastic cases, compared with only 6.3% in medullary and follicular subtypes (P = 0.029).

The medullary subtype was most frequently associated with multisite metastasis (81.2%).

### Ablative techniques

**Figure 1:**
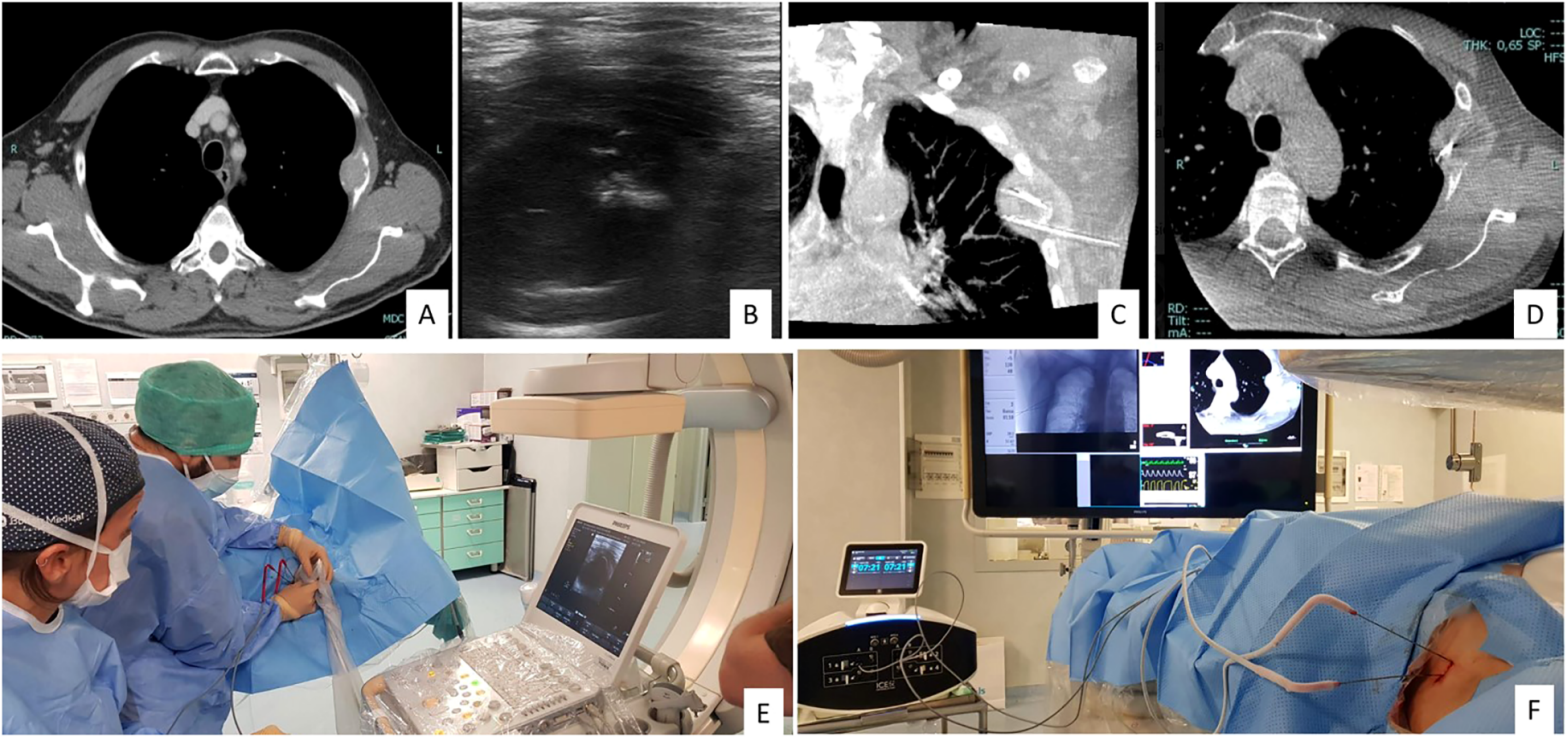
60-year-old man with pleural metastases and osteolytic bone lesion on CT **(A)**. Two antennas for cryoablation (IceCure medical HQ) were placed in the lesion for 4 minutes under US and CBCT guidance **(B-F)**.

Ablative techniques employ thermal energy to induce tumor necrosis through coagulation. In the treatment of liver metastases (LM) from thyroid cancer (TC), the most widely used modalities include radiofrequency ablation (RFA), microwave ablation (MWA), and laser ablation (LA) (4). These procedures—performed percutaneously or laparoscopically—are continuously guided by ultrasound, from electrode (or antenna) placement to energy delivery and post-procedure assessment (**Figure 1**). Ablation is primarily indicated for a limited number of lesions, even when large, for curative intent or tumor debulking, including as a bridge to surgery.

Preprocedural radiological evaluation is critical for determining the number, size, and growth kinetics of LMs, especially in patients with multiple lesions. Lesion location is particularly important: proximity to the biliary ducts—especially near the hepatic hilum—constitutes a relative contraindication due to the risk of ductal stenosis when the bile duct wall lies within the ablation zone (5).

These techniques typically involve short hospitalization and are well tolerated. Potential complications include bile duct injury, hemorrhagic events (e.g. hematoma, intra-abdominal bleeding), liver abscesses, and damage to adjacent organs such as the pleura or lungs (e.g. pleural effusion, pneumothorax).

### Vascular techniques

Vascular interventional radiological techniques (IRTs) involve the intra-arterial administration of therapeutic agents to induce ischemic necrosis, exploiting the typically hypervascular nature of LMs from TC. These techniques vary by the agent used:

- Transarterial embolization (TAE) employs inert embolic agents (e.g., polyvinyl alcohol or microspheres)
- Transarterial chemoembolization (TACE), the most commonly used method, combines embolic agents with chemotherapeutic drugs (e.g., epirubicin)
- Transarterial radioembolization (TARE) uses radioactive microspheres, usually loaded with yttrium-90 (^90Y)

These techniques are primarily indicated for diffuse liver involvement, especially in MTC.

In selected cases, vascular and ablative techniques may be combined, e.g. for devascularising a lesion prior to resection or enhancing local control in large metastases.

Procedure planning typically requires contrast-enhanced CT with a three-phase liver protocol to assess lesion vascularity (**Figure 1**).

Common adverse events include transient elevations in liver enzymes, leukocytosis, and post-embolization syndrome (PES)—characterized by abdominal pain, fever, nausea, and vomiting. Rare but serious events include vascular complications (e.g., bleeding, dissection) and hepatic failure. A potentially better safety profile of TAE compared to TACE remains under investigation (6).

### Evidence from clinical practice

Current evidence on IRT for thyroid cancer LM primarily comes from case reports and small retrospective series, often involving fewer than 50 patients (**Table 1**). Some studies include heterogeneous populations with LM from various primary tumors (e.g. neuroendocrine or hepatic malignancies), limiting the specificity of conclusions for thyroid cancer.

**Table 1:** Published data on interventional radiology treatments for liver metastases from thyroid cancer 9adapted from References (7, 8)].

No. Reference	Author (year)	Type of treatment (No. Patients Histology)	No. (size, range) liver metastases	Treatment response	Adverse events ^
-	*Our series* (2024)	RFA (n. 5– DTC)	Multiple (25–57mm)	PR (n. 2) SD (n. 2) PD (n. 1)	No. 5 ↑liver enzymes (G1), no. 2 tumor necrosis
(8)	*Puleo* (2022)	TARE (n. 8 – MTC)	Multiple (12–551ml)	CR (n. 1) PR (n. 4) SD (n. 1) - (n. 2)	↑liver enzymes (G1-G2), severe liver damage
(7)	*Nervo* et al (2021)	TACE, RFA (n. 3 – MTC, n. 2 – DTC)	Single or multiple (18–55mm)	PR (n. 1) SD (n. 2) PD dopo TACE e PR dopo RFA (n. 1) - (n. 1)	Nausea, abdominal pain, chest pain, ↑liver enzymes (G1-G2)
(9)	*Bergamini* et al (2020)	TACE, TAE, RFA (n. 4 – MTC, n. 2 – DTC)	-	-	-
(10)	*Grozinsky* et al (2017)	TACE (n. 7 – MTC)	Multiple (13–60mm)	PR (n. 7)	Nausea, vomiting, abdominal pain, fever, ↑liver enzymes (G1-G2), hypertensive crisis (G3)
(11)	Akyildiz et al (2010)	RFA laparoscopic (n. 11 – MTC)	-	-	-
(12)	*Mazzaglia* et al (2007)	RFA laparoscopic (n. 9 – MTC)	-	-	-
(13)	*Fromigué* et al (2006)	TACE (n. 12 – MTC)	Multiple (25-98mm)	PR (n. 5) SD (n. 5) PD (n. 2)	Nausea, vomiting, abdominal pain, fever, ↑liver enzymes (G1), tumor necrosis with pain and fever (G3)
(14)	*Lorenz* et al (2005)	TACE (n. 11 – MTC)	Multiple	PR (n. 5) SD (n. 4) PD (n. 1) - (n. 1)	Local erythema, nausea, abdominal pain, ↑hepatic enzymes (G1), hepatic artery dissection (G3)
(15)	*Berber* et al (2002)	RFA laparoscopic (n. 6 – MTC)	-	-	-

^Grade according to Common Terminology Criteria Adverse Events (CTCAE) version 5.0 (16).

PR, partial response.

SD, stable disease.

PD, progressive disease.

MTC, medullary thyroid cancer.

DTC, differentiated thyroid cancer.

RFA, radiofrequency thermal ablation.

TACE, transcatheter arterial chemoembolization.

TAE, transcatheter arterial embolization.

TARE, transcatheter arterial radioembolization.

TRI, interventional radiology treatment.

TACE, RFA, and TARE are the most frequently reported IRTs, with less frequent use of TAE, MWA, and LA. Most treated cases involve MTC.

TACE has demonstrated partial response (PR) rates between 50–100% and long-term disease control (>12 months) in MTC patients (10, 17, 18).

Better outcomes appear associated with lower hepatic tumor burden (<30%), though benefit has also been reported in patients with extensive disease and large metastases (>30 mm) (10).

TACE has also been effective in alleviating symptoms such as diarrhea and abdominal pain.

TACE is generally well tolerated, with adverse events (AEs) being mild and transient. The incidence of AEs increases with repeated procedures, and rare serious complications—such as hypertensive crises or hepatic artery dissection—have been reported.

RFA has shown good results in MTC even for large lesions (up to 70 mm) (19).

In DTC-related LM, data are limited due to the rarity of this presentation. Nonetheless, both vascular and ablative IRTs have been used with encouraging outcomes, including in lesions up to 170 mm in size (20, 21). Reported AEs were typically mild and self-limiting.

IRT has also been successfully integrated into multimodal treatment strategies, including enhancing RAI uptake following local tumor reduction (19, 20), and as neoadjuvant therapy prior to metastasectomy (21).

The combination of IRT with systemic therapies such as TKIs is under preliminary investigation, with early data suggesting potential benefit (4).

### Guidelines

The most recent international guidelines on the treatment of thyroid cancer (TC) acknowledge the use of interventional radiological treatments (IRTs) (**Table 2**) (1, 22, 25, 27–31), although none provide strict recommendations for their application. This is due to both the limited and heterogeneous evidence, much of which is extrapolated from studies on other malignancies, and the high level of technical expertise required to perform these procedures.

**Table 2:** Societal recommendations on the use of Interventional radiologist on the management of metastastic thyroid cancer.

Reference	Guidelines, year	Hystology	Suggestion for interventional radiology treatments
(22)	ATA 2015	DTC	Can be considered valid alternatives to surgery and should be taken into consideration before starting systemic therapy in case of symptomatic metastases/at high risk of local complications
(23)	ATA 2015	MTC	Indicated in patients with growing or symptomatic LM. TACE should be considered in patients with ME <30 mm and limited hepatic involvement (less than one third)
(24)	ESMO 2019	DTC MTC	Can be considered in case of single LM. Interventional treatments may be useful for symptom control in cases of CTM and single dominant LM that are growing compared to the rest of the disease. TACE can be considered in case of contraindication to both surgery and ablative interventional treatments
(25)	ETA 2019	DTC RAI-non response	TACE can be used especially for LM<30 mm and in case of liver involvement <30%. RFA can be used to treat single unresectable LMs or as debulking before surgery
(26)	ETA/CIRS 2021	DTC	Should be considered as part of a multimodal approach in patients at increased surgical risk in whom other treatment modalities would be ineffective

ATA, American Thyroid Association.

CIRS, Cardiovascular and Interventional Radiological Society of Europe.

ESMO, European Society of Medical Oncology.

ETA, European Thyroid Association.

LM, liver metastasis.

RAI, radioiodine.

RFA, radiofrequency thermal ablation.

TACE, transcatheter arterial chemoembolization.

CTD, differentiated thyroid carcinoma.

CTM, medullary thyroid carcinoma.

According to the European Thyroid Association (ETA) guidelines for the management of radioiodine-refractory TC, IRTs may be considered either in combination with systemic therapy or as standalone treatment in cases of single-lesion progression or oligoprogression confined to one organ. The objectives include improving local disease control, delaying the initiation of systemic therapy, and/or relieving symptoms. In this context, tyrosine kinase inhibitor (TKI) therapy can be continued or temporarily paused for a few days (25, 31, 32).

Most guidelines highlight that ablative IRTs should be considered for patients with isolated or limited liver metastases (LMs), particularly when surgical resection is not feasible.

In contrast, vascular IRTs are recommended for patients with diffuse LM, provided that the liver burden is not massive and the lesions are relatively small and accessible. These techniques may also be appropriate when ablative options are contraindicated, such as in cases where metastases are adjacent to the bile ducts or near the hepatic hilum.

All guidelines emphasize the importance of a multimodal, multidisciplinary approach, ideally through discussion in dedicated tumor boards, to ensure that treatment decisions are personalized and aimed at optimizing disease control.

### Clinical benefit and integration in the treatment of thyroid cancer

Interventional radiological treatments (IRTs) such as RFA, MWA, TACE and TARE have shown promise in the treatment of liver metastases (LM) in TC (NEW **Table 3**). However, their clinical benefit is not yet well defined due to limited high-quality data and heterogeneity of patient populations and techniques (25, 31, 32).

**Table 3:** Comparison of interventional radiology techniques for liver metastases in thyroid cancer.

Technique	Indications	Advantages	Limitations	Reported Outcomes
*Radiofrequency Ablation (RFA)*	Few, well localised liver metastases; lesions <5 cm; if surgery is not feasible	Minimally invasive; good local control; repeatable; outpatient treatment possible	Risk of injury to the bile ducts; less effective near large vessels (heat sink effect); size restrictions	Partial response in selected cases; control rates up to 80%; long-term control in small lesions
*Microwave Ablation (MWA)*	Similar to RFA; often preferred for larger lesions or when heat-sink effect is a concern	Faster heating; better penetration; less affected by heat-sink effect; larger ablation zones	Requires specialized equipment; proximity to vital structures is a risk; still heat-sink sensitive	Similar to RFA with potentially larger ablation volumes; promising results in initial studies
*Transarterial Chemoembolization (TACE)*	Diffuse liver metastases, especially in medullary thyroid carcinoma (MTC); symptom control	Combines ischemia with localized chemotherapy; good for hypervascular tumors; symptom relief	Post-embolization syndrome; hepatic toxicity; multiple sessions often required	Partial response rates of 50–100%; symptom control >12 months in some cases
*Transarterial Radioembolization (TARE)*	Diffuse liver involvement, especially when TACE is contraindicated; radioiodine-refractory disease	Delivers targeted internal radiation; useful in poor surgical candidates; prolonged control	Requires radioprotection protocols; limited availability; delayed therapeutic effect	Effective for palliation; improved disease control in selected patients; data still emerging

#### Comparative effectiveness

**Figure 2:**
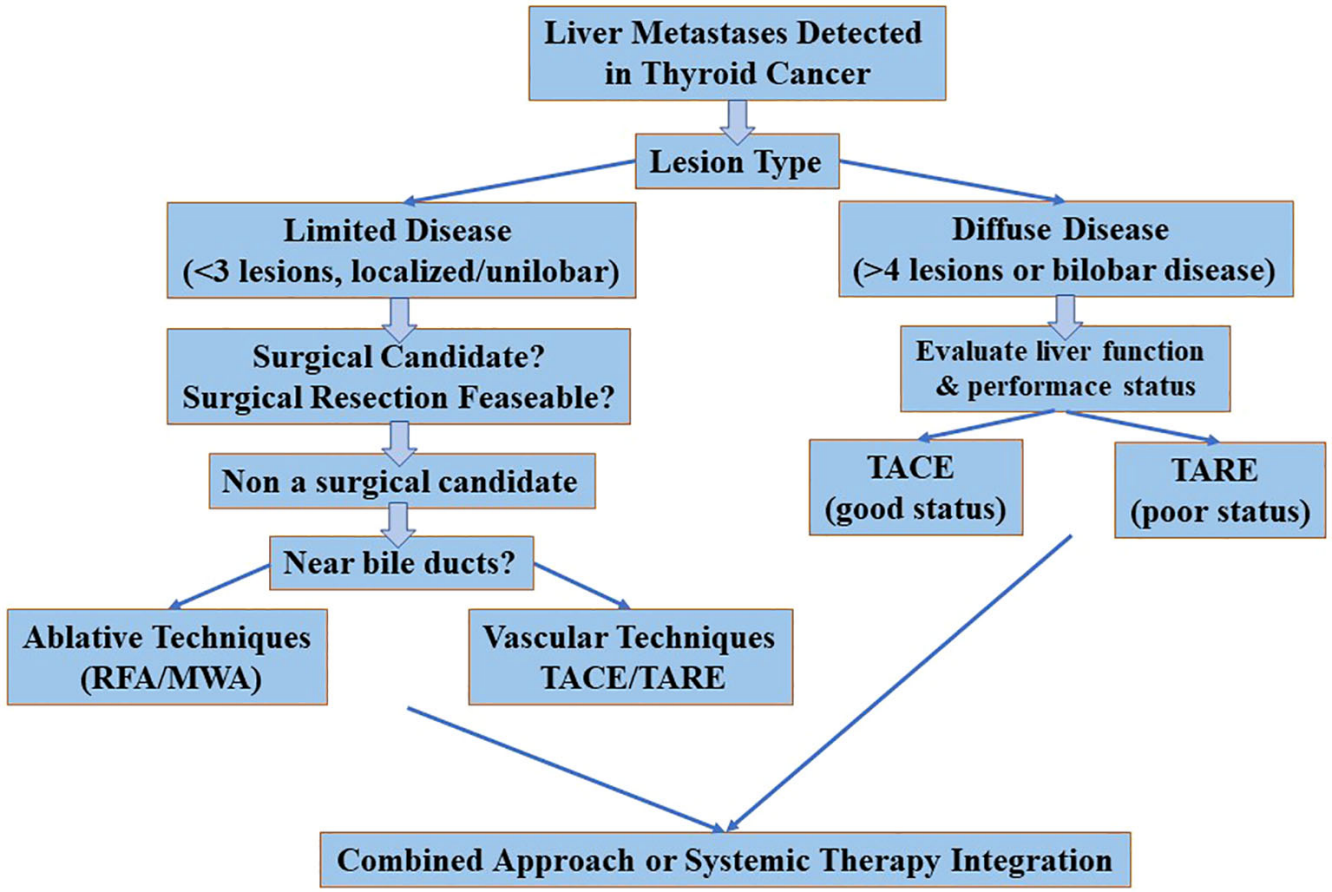
Algorithm for selecting interventional treatments based on clinical scenarios.

Among the ablative techniques, RFA and MWA are the most commonly used. MWA may offer advantages for larger tumors or those close to vascular structures due to its higher and more uniform thermal profile, while RFA remains effective for smaller, peripheral lesions. Laser ablation (LA) has been less frequently reported and its role in LM of TC remains marginal and experimental. Vascular procedures such as TACE and TARE have been shown to be clinically effective, particularly in MTC due to its hypervascular nature (20, 21). TACE is the most commonly described technique, with partial response rates of up to 100% in small series and symptomatic improvement of diarrhoea and abdominal pain. TARE is less well studied but may be an alternative for patients who are not suitable for TACE or have larger tumors. There is a lack of comparative data and no head-to-head studies have been conducted. Consequently, the choice of technique still depends on availability, the experience of the surgeon, the characteristics of the lesions (size, location, number) and vascularity (**Figure 2**).

#### Criteria for patient selection

Patient selection is crucial for the success of IRT (19, 21, 30). Candidates for ablative IRT usually have:

- A limited number of LMs (usually ≤3)
- A good performance status
- Absence of lesions near critical structures (e.g. bile ducts)
- Stable or indolent extrahepatic disease

Vascular IRTs (e.g. TACE/TARE) are more suitable for:

- Multiple or bilobar liver metastases
- Predominantly MTC histotype
- Symptomatic disease requiring rapid tumor control
- Patients who are not candidates for surgery or systemic therapies

Careful multidisciplinary assessment is essential, involving endocrinologists, interventional radiologists, oncologists and surgeons, to determine the best therapeutic strategy.

#### Limitations and safety profile

The main limitations of IRTs in TC are:

- Lack of prospective data
- Risk of complications (e.g. bile duct injury, liver abscess, post-embolization syndrome)
- Difficulty in treating lesions near the hilum
- Possible overlap or interference with systemic therapies (e.g. anti-angiogenic TKIs)

Most reported adverse events are mild and transient, but serious events such as hepatic artery dissection or liver failure may occur, albeit rarely (6, 30).

#### Long-term outcomes and survival benefit

Data on long-term outcomes are sparse. Retrospective series report durable local control (>12 months in many cases) and symptom relief, particularly in MTC (30, 32). However, there is no definitive evidence that IRT improves overall survival and it should currently be considered as part of a palliative or adjunctive approach rather than a curative measure.

#### Integration into clinical pathways

International guidelines now cautiously refer to IRT as an optional treatment for oligoprogressive disease or as a means of delaying the initiation of systemic therapy in RAI-refractory DTC or metastatic MTC (25, 31, 32). In practice, however, IRT is only used in large centers with interventional expertise and is often outside the standardized treatment pathways. To better integrate IRTs into the treatment of thyroid cancer, future steps should include:

- Prospective registries and multi-center studies
- Inclusion of IRT in national and international guidelines
- Definition of standard referral pathways and criteria for patient authorization

In summary, while IRT is a valuable therapeutic option for selected patients, its role needs to be defined as part of a multimodal and personalized treatment strategy supported by structured evidence and multidisciplinary leadership.

### Biomarkers for non-invasive detection and monitoring

The integration of biochemical and molecular biomarkers with radiological techniques offers promising opportunities to improve the detection, monitoring and personalized treatment of LM in thyroid cancer TC. While IRTs allow local control, non-invasive biomarkers can serve as valuable tools for early detection of metastatic progression, assessment of response to treatment and follow-up after surgery (33–35). In MTC, calcitonin and carcinoembryonic antigen (CEA) are well-established circulating biomarkers. A progressive increase in calcitonin, especially with a doubling time of less than 6 months, is a highly predictive indicator of metastatic disease, including liver. CEA can complement calcitonin by providing additional prognostic information, especially in de-differentiated or rapidly progressing disease. High baseline levels of calcitonin, often above 1,000 pg/ml, have been associated with systemic spread and should prompt more intensive radiological monitoring, particularly of the liver. A decrease in calcitonin levels or stabilization of its doubling time after IRT may indicate a response to treatment and contribute to early assessment of therapeutic benefit, particularly if radiological changes are delayed or equivocal. In differentiated thyroid carcinoma, serum thyroglobulin (Tg) is the most important biomarker for monitoring recurrence or metastasis in patients treated with total thyroidectomy and radioiodine ablation. In patients with radioiodine-refractory disease, an increase in unstimulated Tg may precede radiological evidence of LM and should be considered an early signal for intensification of imaging and re-evaluation of therapeutic strategies (33–38). Following IRT, Tg kinetics may be useful in monitoring response, although fluctuations due to tumor necrosis or inflammation should be interpreted with caution. In both DTC and MTC, the absence of a corresponding increase in tumor markers despite radiological progression, or vice versa, may indicate tumor dedifferentiation requiring biopsy, molecular profiling or a change in therapeutic strategy.

#### Emerging biomarkers and molecular tools

Advances in liquid biopsy, including the detection of circulating tumor DNA (ctDNA) and microRNAs (miRNAs), may provide future opportunities for non-invasive, real-time monitoring of metastatic burden. These tools could potentially:

- Detect minimal residual disease after local treatment (e.g. RFA or TACE)
- Identify resistance mutations under TKI therapy
- Stratify patients who are likely to benefit from IRT compared to systemic approaches

Although not yet routinely used in thyroid cancer, ongoing studies are investigating their feasibility and predictive value, particularly in advanced or treatment-resistant cases.

#### Multimodal integration

The combination of biochemical markers with radiological examinations improves the precision of patient selection for IRTs and enables more individualized follow-up strategies:

- High-risk biomarker profiles (e.g. rapidly rising calcitonin or Tg) can prioritize patients for early IRT.
- Post-intervention biomarker trends can be used to help decide on the timing of systemic therapy or repeated local interventions.

**Figure 3:**
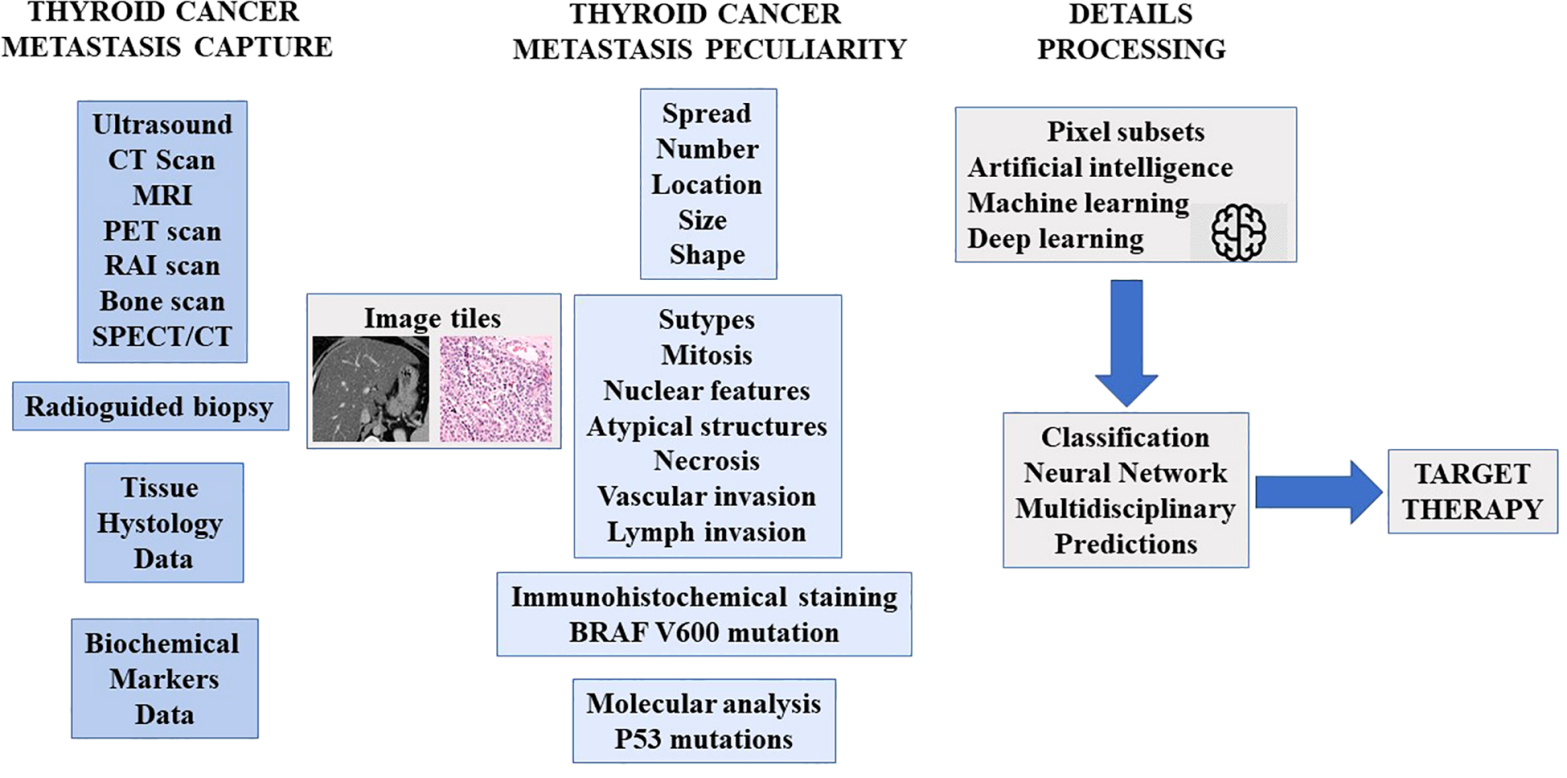
Proposed integration of radiological and biochemical markers for the assessment of metastatic disease.

Future clinical algorithms should incorporate both imaging and biomarkers for comprehensive disease monitoring to ensure timely therapeutic adjustments and optimize outcomes (**Figure 3**).

## Discussion

The use of IRT in the treatment of LM in TC is a promising but under-researched therapeutic option. While both ablative and vascular techniques have shown encouraging results in selected cases, current evidence is mainly based on small, retrospective studies or single case reports with limited generalizability. Ablative techniques such as RFA, MWA and LA appear to be particularly suitable for patients with a limited number of LM, especially when the lesions are well localized and surgically inaccessible. These procedures provide good local control, even in large metastases, and are generally well tolerated. However, their applicability is limited by technical considerations — in particular the proximity of the lesions to critical biliary structures — and patient-specific factors. Despite promising results, the heterogeneity of techniques and reporting standards limits the ability to draw robust conclusions regarding efficacy or comparative superiority.

Vascular techniques, including TACE, TAE and TARE, have been used more frequently in MTC due to the typically hypervascular nature of these metastases. TACE in particular has shown partial response rates of up to 100% in some small series and can help to alleviate symptoms such as diarrhoea or pain. However, these procedures are associated with a unique toxicity profile — including post-embolization syndrome and, less commonly, vascular complications — and require careful patient selection.

The combined use of ablative and vascular IRTs as part of a multimodality treatment strategy has been proposed in certain clinical contexts, such as debulking prior to systemic therapy or to enable metastasectomy. In addition, some reports suggest a potential role for IRT in improving the efficacy of radioiodine therapy or maintaining disease control during treatment with tyrosine kinase inhibitors (TKIs). Although these applications are theoretically sound, they have not been validated in prospective studies and are based on highly selected populations.

A major limitation of the current literature is the lack of prospective data, standardized outcome reports and uniform approval criteria. In addition, many reports include patients with liver metastases that are not due to thyroid cancer, making it difficult to limit the results specifically to thyroid cancer. The rarity of liver metastases in DTC and the often widespread disease in MTC further complicate a systematic review.

Guidelines from the European Thyroid Association and other societies now cautiously recognize IRT as a viable option in the treatment landscape of RAI-refractory or oligoprogressive TC, but there are no standardized algorithms. Decision-making remains highly individualized and should be carried out in a multidisciplinary setting, ideally with the involvement of dedicated endocrine tumor boards.

In addition to imaging and IRT techniques, the inclusion of biochemical and molecular markers in the clinical decision-making process can improve patient stratification and treatment monitoring. Biomarkers related to oxidative stress, as well as new genomic and proteomic profiles, are increasingly being investigated for their prognostic and predictive utility in advanced thyroid cancer. These could help identify patients at higher risk of metastasis, including the liver, and guide the intensity or combination of treatments.

In addition, liquid biopsy techniques — such as circulating tumor DNA (ctDNA), circulating tumor cells (CTCs) and exosomal RNA profiles — hold promise for non-invasive detection of micrometastases. In thyroid cancer, preliminary studies have shown the feasibility of liquid biopsy for monitoring disease burden, predicting response to treatment and detecting early recurrence, although clinical implementation is still in its infancy. Incorporating these approaches into future studies with IRT may allow for better response assessment and long-term monitoring in patients with liver metastases.

### Knowledge gaps and future directions

A key limitation of the current literature is the lack of prospective data, standardized outcome reports and uniform approval criteria. In addition, many reports include patients with liver metastases that are not due to thyroid cancer, making it difficult to limit the results specifically to thyroid cancer. The rarity of liver metastases in DTC and the often widespread disease in MTC further complicate systematic investigation.

- Lack of high-level evidence: There is an urgent need for multicenter prospective studies to evaluate the efficacy and safety of IRTs in well-defined TC populations.
- Definition of patient selection criteria: Stratification by tumor burden, location, histotype and previous treatments is essential to optimize outcomes.
- Standardization of outcome measures: The use of RECIST criteria, symptom relief scales and quality of life metrics should be encouraged to allow comparison between trials.
- Integration with systemic therapies: The timing and sequencing of IRT in relation to TKIs is still uncertain and requires further clinical investigation.
- Cost-effectiveness and access: Assessing the feasibility and sustainability of IRT in different healthcare settings should be part of future health policy considerations.

In summary, while IRTs show promise for the treatment of LM in TC, their current use is largely empirical and limited to specialist centers. Moving this area forward requires not only better evidence, but also consensus on clinical pathways to support consistent, evidence-based decision-making.

## Conclusions

Vascular and ablative interventional radiological treatments (IRTs) are a promising option for improving disease control or palliation in patients with liver metastases (LM) from medullary thyroid carcinoma (MTC) and differentiated thyroid carcinoma (DTC), although current evidence is limited. These procedures are generally well tolerated and can be used either as stand-alone measures or in combination with each other or with systemic therapies (e.g. tyrosine kinase inhibitors [TKIs]) as part of a multimodal treatment strategy. It is crucial that IRTs are carried out in specialized centers with extensive expertise and only after rigorous patient selection in a multidisciplinary setting. Prospective, multicenter studies are urgently needed in advanced thyroid cancer (TC) to better define patient selection criteria and optimal timing of treatment and to determine the true impact of IRTs on long-term survival outcomes (39–41).
